# Cytomegalovirus-Associated Splenic Infarction in a Young Female Patient

**DOI:** 10.7759/cureus.10801

**Published:** 2020-10-05

**Authors:** Mayurathan Pakkiyaretnam, Christoper Horne, Louise Sarr, Meenakshi Parsad, Jimmy Chong

**Affiliations:** 1 Diabetes and Endocrinology, Royal Hampshire County Hospital, Winchester, GBR

**Keywords:** cytomegalovirus, splenic infarction

## Abstract

Cytomegalovirus (CMV) infection generally causes asymptomatic infection in the majority of immunocompetent individuals. However, the presentation may be complicated by life-threatening conditions in immunocompromised patients. We report a case of a 23-year-old healthy Caucasian female with acute CMV infection and splenic infarction. Serological studies confirmed acute CMV infection, and echocardiography did not show any evidence of endocarditis or mural thrombosis. We did not consider antiviral and anticoagulation therapies due to the immunocompetent nature of the patient and since the condition was suspected to be a minor vessel disease likely triggered by CMV infection.

## Introduction

Cytomegalovirus (CMV) is a common and harmless virus and it belongs to the Herpesviridae family of viruses. Acute infection usually causes asymptomatic infection in immunocompetent individuals. CMV spreads from person to person by saliva and infected body fluids including blood, semen, tears, breast milk, and urine. Common symptoms of CMV infection are fever, muscle pain, fatigue, and sore throat. Occasionally lymphadenopathy and hepatosplenomegaly can be detected on examination. However, it can cause significant morbidity and mortality among pregnant and immunocompromised patients [1]. Immunocompetent patients with CMV infection do not require any antiviral treatment. Valganciclovir is the popular antiviral agent to treat CMV infection in immunocompromised patients. Serious complications of vasculopathy and thrombosis associated with CMV infection have been reported extensively in immunocompromised patients [2]. However, splenic infarction in immunocompetent patients is uncommon [3,4].

## Case presentation

A previously healthy 23-year-old Caucasian female presented with a one-week history of headache and five days of high-grade fever. The headache was of gradual onset, generalized, constant, and aching in nature, associated with photophobia and neck stiffness. There were no alteration in the level of consciousness, seizures, or focal neurological features. Two days later, she developed severe body aches and pains. The patient did not have a sore throat, earache or discharge, cough, chest pain, palpitations, or shortness of breath. She did not complain of loss of appetite, weight loss, diarrhea, abdominal pain, or any urinary symptoms. There were no rashes or clinical evidence of arthritis. She had not been in recent contact with animals or ill people. She had a past medical history of well-controlled asthma, and there was no history of any thrombotic episodes or recurrent miscarriages. She was a teetotaller and not into intravenous or any other form of recreational drug use.

On examination, her Glasgow Coma Scale (GCS) was 15 and the oral temperature was 38.3 ºC on admission. She had mild neck stiffness, but Kernig’s sign was negative. There were no rashes and no focal neurological signs, and the examination of optic fundi was normal. Her respiratory and cardiovascular system examinations were also normal. Abdominal examination revealed mild splenomegaly and normal bowel sounds without hepatomegaly or ascites.

Meningitis was the initial clinical diagnosis on admission, and she was started on acyclovir and ceftriaxone. Her CT scan of the brain was normal. Other initial laboratory investigations revealed a white blood cell (WBC) count of 8.3 x 10^9^/L with 4.53 x 10^9^/L of lymphocytes. Lymphocytosis with atypical and reactive lymphocytes was observed in the blood film. Elevated alanine transaminase (ALT) levels of 510 U/L and alkaline phosphatase (ALP) levels of 122 were also detected. Multiple attempts for lumbar puncture including by the anesthetic team were unsuccessful.

**Figure 1:**
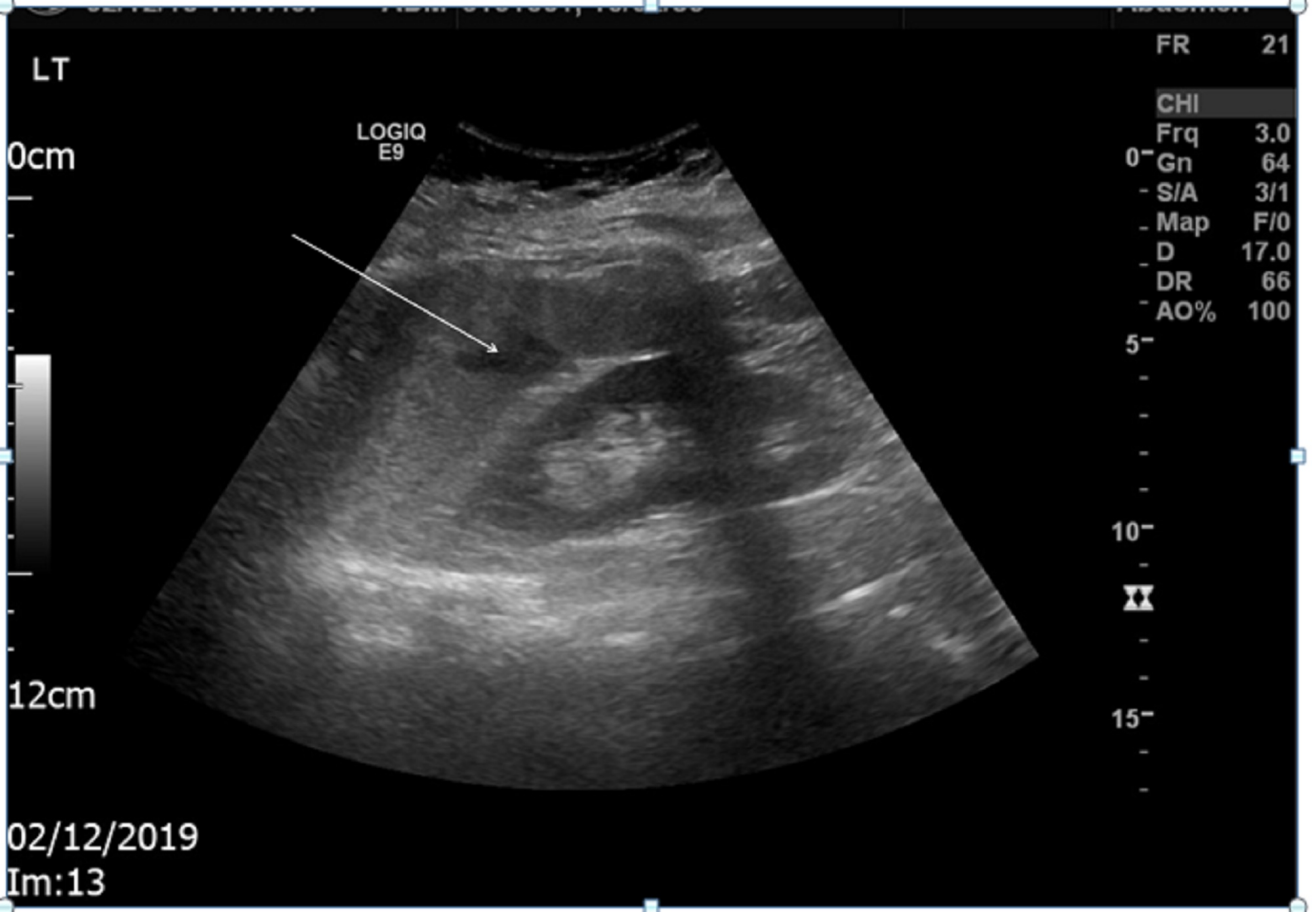
Abdominal ultrasound of the patient Splenic infarction is indicated by the arrow

An abdominal ultrasound scan showed splenomegaly of approximately 15.3 cm in bipolar length with a small hypoechoic area in the spleen parenchyma suggestive of small splenic infarct (two consultant radiologists discussed and confirmed the same). There were no other ultrasonically evident abnormalities identified, especially no thrombosis of the splenic artery, splenic vein, or hepatic vein (Figure 1).

Her trans-thoracic echocardiogram showed normal ejection fraction and filling, and there were no valvular lesions, vegetations, or mural thrombosis. Both her blood and urine cultures were sterile. The patient’s test results on admission, on the fifth day, on discharge, and at the two-week follow-up are summarized in Table 1.

**Table 1:** Summary of basic blood investigations *Indicates reference range eGFR: estimated glomerular filtration rate; CKD-EPI: Chronic Kidney Disease Epidemiology Collaboration

Variables	On admission	Fifth day of admission	On discharge (13th day of admission)	Two weeks after discharge
Hemoglobin (120–160)* g/L	129	143	110	131
White blood cells (WBC) (4–11)* x 10^9^/L	8.3	8.8	8.4	7.5
Neutrophils (1.5–8)* x 10^9^/L	3.04	3.26	2.44	2.70
Lymphocytes (1.3–4)* x 10^9^/L	4.52	3.78	4.28	3.60
Monocytes (0.2–0.8)* x 10^9^/L	0.00	0.53	0.67	0.45
Eosinophils (0–0.8)* x 10^9^/L	0.66	0.70	0.34	0.30
Basophils (0–0.3)* x 10^9^/L	0.00	0.18	0.17	o.15
Platelets (150–450)* x 10^9^/L	258	253	255	283
C-reactive protein (CRP) (0–5)* mg/L	75	34	13	11
Alanine transaminase (ALT) (10–49)* U/L	510	430	123	52
Alkaline phosphatase (ALP) (46–116)* U/L	122	175	80	69
Bilirubin (5–21)* µmol/L	13	7	8	9
Albumin (34–50)* g/L	32	35	30	36
Prothrombin time (PT) (12.5–14.4)* seconds	15.4	-	14.1	13.6
Activated partial thromboplastin time (aPTT) (24.5–37.1)* seconds	35.8	-	38.8	48.9
Fibrinogen (1.6–4.2)* g/L	4.3	-	3.8	3.5
Sodium (133–146)* mmol/L	136	139	139	137
Potassium (3.8–5.3)* mmol/L	3.8	4.2	4.0	4.7
Urea (2.5–7.8)* mmol/L	2.9	3.7	2.7	3.6
Enzymatic creatinine (45–84)* µmol/L	83	64	68	68
eGFR (CKD-EPI equation) (60–99999)* mls/min	86	>90	>90	>90
Corrected calcium (2.2–2.62)* mmol/L	2.21	-	-	-
Phosphate (0.8–1.5)* mmol/L	1.38	-	-	-

Subsequently, her Paul-Bunnell test and pneumococcal and meningococcal antigen/polymerase chain reaction (PCR) were negative. Her hepatitis B surface antigen, hepatitis C antibody, HIV 1 and 2 antigen/antibody, Mycoplasma immunoglobulin M (IgM), Leptospira IgM/Lipl32 DNA/16S rRNA, Coxiella IgM/IgG/PCR, Toxoplasma antibody, and IgM/IgG antibodies for Lyme disease were negative. IgM and IgG for CMV and Epstein-Bar virus (EBV) were positive initially. However, EBV PCR was negative, and CMV PCR quantification demonstrated 920 DNA copies/ml of CMV. A microbiologist confirmed acute CMV infection.

At this point, acyclovir and ceftriaxone were discontinued as she was immunocompetent. Her prothrombin time (PT) was normal. But she had a raised activated partial thromboplastin time (aPTT) of 38 8 and 48.9 seconds (reference range: 24.5-37.1 seconds). Antiphospholipid antibodies (APA) showed positive lupus anticoagulant [Dil viper venom time (DRVT) of 1.29; reference range: 0.85-1.15] and negative IgM and IgG anticardiolipin antibodies. The positive lupus anticoagulant was likely due to the CMV infection. Therefore, anticoagulation therapy was not considered as the condition was suspected to be a minor vessel disease likely triggered by CMV infection.

Later, she was discharged on the 13th day without any specific medications. On discharge, we arranged an out-patient clinic follow-up to evaluate her clinically and biochemically. In two weeks’ time, she was well and almost all of her blood investigations were back to normal. She had slightly raised aPTT. The thrombophilia team had advised that she did not need any further investigations at this point and there was no need to repeat the antiphospholipid antibodies. However, it was conveyed that she should be evaluated in detail, including repeating antiphospholipid antibodies, and should be considered for anticoagulation if she developed any further thrombotic episode in the future. All of this information and lifestyle modifications were explained to the patient, especially about danger signs and follow-up plan upon discharge.

## Discussion

We reported the case of a young healthy immunocompetent female with acute CMV infection and splenic infarction. Initially, empirical treatment for meningitis was initiated because of the clinical picture; however, the antibiotics and antiviral treatments were discontinued with the diagnosis of CMV infection. Acute CMV infection and splenic infarction are rare in immunocompetent patients, and only a few case reports have been reported in the literature [2,3,4,5]. The exact pathophysiological mechanism by which CMV infection triggers thrombosis is unclear. However, several theories suggest that CMV induces thrombosis by activating platelet and leukocyte adhesion to infected endothelial cells, increasing the levels of factor VIII and vascular smooth muscle proliferation. It is also known to increases platelet-derived growth factor, transforming growth factor-β, interleukin (IL)-1β, IL-6 and tumor necrosis factor (TNF)-α, and cryoglobulinemia. The most commonly accepted theory describes a transient increase in antiphospholipid antibodies due to acute CMV infection. As a result, CMV infection can induce inflammation and thrombosis either due to one or a combination of the above-mentioned mechanisms [2,6]. Interestingly, CMV can cause venous as well as arterial thrombosis [6]. There have been reports describing deep vein thrombosis and life-threatening thrombosis, such as pulmonary embolism, hepatic and portal vein thrombosis, renal artery thrombosis, and myocardial infarction [3,4,6].

Infections associated with splenic infarction can be seen in endocarditis, malaria, and viral infections such as CMV, parvovirus B19, EBV and human herpesvirus type 6, and HIV [5,7]. Sometimes, there may be a cross-reaction and false-positive EBV immunoglobulins in CMV infections [1]. A clear diagnosis could be obtained using nucleic acid amplification tests by doing PCR studies.

Antiviral treatment for acute CMV infection is indicated for immunocompromised patients and occasionally due to severe multi-organ involvement in immunocompetent patients [4]. Anticoagulation therapy is not advised for immunocompetent patients with minor diseases. However, some patients have been anticoagulated due to the presence of congenital hypercoagulable conditions [2]. The duration of anticoagulant therapy has ranged between a few weeks to one year, but it is not clearly mentioned in most of the reports [6].

## Conclusions

Acute CMV infection with various thrombotic manifestations has been reported in the medical literature. Anyone presenting with unexplained fever and arterial or venous thrombosis should be ideally investigated for CMV infection. If CMV infection is confirmed in a patient with arterial or venous thrombosis, full thrombophilia screening would be beneficial to decide about the need for anticoagulation treatment and its duration. Moreover, each and every case of CMV infection with thrombosis should be evaluated individually to determine the need for antiviral and anticoagulation therapies.
